# Antidepressant Discontinuation Syndrome in Patients on SSRIs During Viral Infections: A Study of Prevalence and Mechanisms

**DOI:** 10.1192/j.eurpsy.2025.1800

**Published:** 2025-08-26

**Authors:** M. S. Artemieva, I. E. Danilin, A. T. M. Mkrtchyan, L. D. Osiia

**Affiliations:** 1 Psychiatry and medical psychology, RUDN University, Moscow, Russian Federation

## Abstract

**Introduction:**

This study examines the frequency and underlying mechanisms of antidepressant discontinuation syndrome (ADS) in patients receiving SSRI therapy who subsequently experience viral infections such as influenza or COVID-19. Clinical observations suggest that viral infections may diminish the efficacy of SSRIs, potentially triggering ADS even without changes in dosage.

**Objectives:**

To assess the prevalence of ADS symptoms among patients undergoing SSRI treatment during viral infections and to explore potential biological mechanisms behind this interaction.

**Methods:**

A sample of 84 individuals was recruited from a large Russian-language forum for users of antidepressants. Data on ADS symptoms onset and exacerbation during viral infections were collected using a survey based on the DESS scale. Inclusion criteria for ADS were the new appearance or worsening of withdrawal symptoms specifically during the viral illness.

**Results:**

A significant portion of the cohort reported ADS symptoms. Key findings included dizziness (62%), increased anxiety (40%), mood instability (57%), sleep disturbances (65%), and memory impairment (40%). Notably, brain zaps were observed in 63% of patients, identifying this symptom as distinct to ADS rather than typical viral symptomatology.

**Conclusions:**

The study indicates a high prevalence of ADS symptoms in patients continuing SSRI therapy during viral infections, likely influenced by neuroinflammatory responses and cytokine release, along with tryptophan depletion driven by interferon activity. These insights underscore the importance of vigilance and potentially adapting SSRI management strategies during periods of viral illness to mitigate ADS effects.

**Disclosure of Interest:**

None Declared

